# Socioeconomic Impact of Recurrent Primary Spontaneous Pneumothorax: Should Video-Assisted Thoracoscopic Surgery Be Considered at First Episode of Primary Spontaneous Pneumothorax?

**DOI:** 10.3390/healthcare9091236

**Published:** 2021-09-19

**Authors:** Stephen Fung, Andrea Alexander, Hany Ashmawy, Levent Dizdar, Sami Safi, Alexander Rehders, Georg Fluegen, Wolfram Trudo Knoefel

**Affiliations:** Department of Surgery, University Hospital Duesseldorf and Heinrich-Heine-University Duesseldorf, Moorenstrasse 5, 40225 Duesseldorf, Germany; stephen.fung@med.uni-duesseldorf.de (S.F.); Andrea.Alexander@med.uni-duesseldorf.de (A.A.); Hany.Ashmawy@med.uni-duesseldorf.de (H.A.); Levent.Dizdar@med.uni-duesseldorf.de (L.D.); Sami-Alexander.Safi@med.uni-duesseldorf.de (S.S.); Rehders@med.uni-duesseldorf.de (A.R.); Georg.Fluegen@med.uni-duesseldorf.de (G.F.)

**Keywords:** primary spontaneous pneumothorax, recurrence, socioeconomic impact

## Abstract

Background: Current guidelines recommend video-assisted thoracoscopic surgery (VATS) for recurrent primary spontaneous pneumothorax (PSP) and for cases with persistent air leak after chest tube treatment. The socioeconomic impact of recurrent PSP on the healthcare system is insufficiently reported. Methods: Ninety-six patients treated for PSP between 01/2010 and 01/2020 were included. Forty-eight patients underwent primary VATS, while the second group received chest tube (CT) treatment only. Length of hospital stay (LOS), duration of chest tube, prolonged air leak, postoperative complications, recurrences and treatment costs were analyzed. Results: Prolonged air leaks were evident in 12.5% and 22.9% patients of the VATS and CT group, respectively. Ten (20.8%) patients in the CT group underwent VATS for persistent air leakage. During follow-up, the VATS group recurred at 8.3% compared to 52.1% in the CT group. The total cost of treatment per patient, including treatment cost due to recurrence, was EUR 1.501 in the VATS group and EUR 2.233 in the CT group. Conclusions: Primary treatment of PSP by CT is associated with an increased socioeconomic burden for patients and the healthcare system due to high recurrence rates. This burden may be reduced if VATS is considered at the first episode of PSP.

## 1. Introduction

According to the German S3 guidelines [1], primary spontaneous pneumothorax (PSP) describes the presence of air without preceding trauma or underlying pulmonary disease within the pleural space of patients under 45 years of age. The incidence of PSP has been reported as approximately 1–9.8 and 7–24 cases per 100,000 individuals per year in females and males, respectively [2,3]. According to the current guidelines [1,4,5,6], the initial treatment algorithm depending on the patient’s clinical condition includes observation, oxygen supplementation and chest tube drainage. Although PSP often resolves by chest tube drainage, high rates of recurrence after this treatment have been described [7,8,9,10]. In cases with ipsilateral or contralateral recurrence of PSP and for those with persistent air leak following chest tube (CT) treatment, the guidelines [1,4,5,6] recommend video-assisted thoracoscopic surgery (VATS). In various studies, VATS bullectomy with or without pleurectomy was associated with very low rates of recurrence and a short length of hospitalization, even at first episode of PSP [10,11,12,13,14]. Therefore, we wondered if preventive surgical intervention might be a suitable alternative to reduce the recurrence rates and associated economic burden, as well as to mitigate the negative psychological impact that results from anxiety about a potential recurrence [15]. To date, the economic and social impact due to hospital readmission for recurrent PSP on both patients and on the healthcare system is scarcely elucidated. Thus, the aim of this study was to retrospectively analyze the socioeconomic impact of recurrent PSP following VATS and CT treatment in our institution.

## 2. Materials and Methods

We retrospectively reviewed the data of 96 patients with PSP treated in our institution either by CT or VATS between January 2010 and January 2020. Patients’ clinical data, including age, gender, weight, height, body mass index (BMI), length of hospital stay (LOS), duration of chest tube, postoperative complications and length of air leakage were retrieved from the medical records. One group underwent immediate surgery (VATS) (39 males and 9 females) during primary hospitalization; the other group received CT treatment only (40 males, 8 females). The mean age of the patients treated using VATS was 24.8 years (range 18–39), and was 26.2 years (range 18–40) for those who underwent CT treatment.

Patients in the VATS group underwent immediate surgery (these patients underwent VATS after a mean period of 2 days (range 1–4 days) after presentation in our emergency room upon first hospitalization) due to the following conditions: occurrence of second ipsilateral or contralateral pneumothorax (recurrence) (*n* = 38), synchronous bilateral spontaneous pneumothorax (*n* = 6) and spontaneous hemopneumothorax (*n* = 4). Patients in the CT group received chest tube drainage only upon first episode of unilateral PSP. Patients initially planned for CT treatment but suffering prolonged air leak (>5 days) underwent secondary VATS during first hospitalization. Although secondary VATS was performed, we assigned these patients for comparability to the CT group based on their initial treatment. In both groups, low-dose computer tomography of the lungs was performed prior to surgery to detect any bullous disease.

Patients who relapsed after CT treatment or VATS underwent VATS or re-VATS, respectively. After discharge, all patients were closely monitored for complications as outpatients. Follow-up consisted of 3-monthly consultations for one year. For long-term follow-up, the patients were contacted and assessed with a questionnaire. For all patients, the mean follow-up period was 46.2 months (range 1–119).

The local Institutional Review Board of the Heinrich Heine University Clinic of Duesseldorf approved this study (Study Nr: 2020-1271).

### 2.1. Surgical Procedure—VATS

All patients underwent the same surgical treatment, consisting of partial pleurectomy and bullectomy when blebs were evident. VATS was performed under general anesthesia with double-lumen tube intubation and single-lung ventilation. After the lateral positioning of the patient, VATS was performed by the conventional two- or three-port approach. Following initial thoracoscopy and thorough inspection of the visceral and parietal pleura, bullectomy was carried out where blebs or bullae were identified by wedge resection using an endoscopic stapling device (Autosuture GIA Universal; Covidien, Mansfield, MA, USA). Partial pleurectomy was performed in all patients beginning from the apex of the pleura cavity up to the 7th or 8th intercostal space. An underwater air leak test was performed to verify the lack of residual air leaks. One 24 French (Fr) chest tube (COVIDIEN^TM^) placed at the apex of the thorax cavity was inserted through the trocar incisions at the 5th intercostal space. The chest tube was connected to a digital chest drainage system (Thopaz+, Medela AG, Baar, Switzerland) with a suction of −20 cmH_2_O.

### 2.2. Chest Tube (CT) Treatment

In the CT group, one chest tube drainage (COVIDIEN^TM^, 24 Fr) was used. This was inserted under local anesthesia by thoracostomic access in the mid axillary line at the level of the 4th or 5th intercostal space. The chest tube was connected to a digital chest drainage system (Thopaz+, Medela AG, Baar, Switzerland) with a suction of −20 cmH_2_O.

### 2.3. Statistical Analysis

Simple descriptive statistics were used. Data were expressed as mean value, range and percentages. Mean values of continuous variables between groups were compared with a Mann–Whitney U test and categorical variables with a chi-square test. Statistical significance was considered at *p* < 0.05. Statistical analysis was performed in Microsoft Excel and SPSS 25.0 (IBM Corp, released 2017. IBM SPSS Statistics for Windows, Version 25.0., IBM, Armonk, New York, NY, USA).

## 3. Results

Ninety-six PSP patients were included in this study, of whom 48 patients underwent primary VATS and 48 patients received CT treatment. Table 1 shows patients’ demographic data. None of the patients were lost during follow-up. Patients treated by thoracotomy, needle aspiration or observation were initially excluded from this study.

For both groups, the following parameters were analyzed from the medical records.

### 3.1. Length of Hospital Stay (LOS)

The mean LOS during primary hospitalization was 6 days in both groups. After VATS for recurrence, the mean LOS of the second hospitalization was also 6 days (Table 2 and Table 3).

### 3.2. Duration of Chest Tube Drainage

Duration of chest tube drainage was shorter in the VATS group (5 days) compared to the CT group (6 days). After VATS for recurrence, the mean duration of chest tube drainage in both groups was 5 days (Table 2 and Table 3).

### 3.3. Treatment of Complications

One patient (2.1%) in the VATS group developed postoperative hemothorax and was reoperated on by VATS. Air leaks were considered as prolonged when evident for more than 5 days after treatment (VATS or CT). In the VATS group, six (12.5%) patients had persistent air leak, which resolved spontaneously on postoperative day 6 or 7. Prolonged air leak was also observed in 11 (22.9%) patients of the CT group. Ten (20.8%) patients of this group underwent VATS and one resolved spontaneously on postoperative day 8 (Table 2).

### 3.4. Management Costs

As defined in the German diagnosis-related groups (DRG) catalogue, the cost of a hospital stay per day for patients with PSP is calculated at EUR 148. The cost of surgical materials for VATS or chest tube placement under local anesthesia is EUR 465 and EUR 77, respectively (Table 4).

One patient in the VATS group underwent re-VATS for hemothorax and 10 patients in the CT group underwent VATS due to prolonged and persistent air leakage. The costs of treatment for these 10 patients were assigned to the CT group and not to the VATS group due to their initial treatment. The combined cost of treatment for these cases was EUR 1353 for the VATS group and EUR 13,530 for the CT group (Table 5). With a mean hospital stay of 6 days in both groups (*p* = 1.00), the total treatment cost (including material, hospitalization and complication treatment costs) prior to recurrence, excluding additional medication for comorbidities, was calculated at EUR 1.360 and EUR 1.247 per patient for the VATS and CT group, respectively (Table 4).

During a mean follow-up period of 46.2 months, 4 (8.3%) and 25 (52.1%) patients of the VATS and CT group presented with recurrent PSP (*p* < 0.001), respectively. All patients underwent re-VATS or VATS, according to the previous treatment, incurring a further cost of EUR 1.353 per patient (Table 3). Including treatment cost for recurrence, the total management cost per patient was EUR 1.473 in the VATS group and EUR 1.952 for the CT group (Δ = EUR 479, Table 6). The total management cost increased to EUR 1.501 and EUR 2.233 per patient in the VATS and CT group, respectively, after adding treatment costs for complications (Δ = EUR 732; Table 6).

## 4. Discussion

To date, chest tube drainage is still a recommended treatment for the first episode of PSP. According to the current guidelines [1,4,5,6], VATS should be considered at recurrence or in case of persistent air leak after CT treatment. Similar to our results, various studies have proven VATS to be superior to CT treatment in terms of recurrence rates, even at first episode of PSP [12,13,14]. VATS has also been reported to be associated with shorter hospitalization rates and better quality of life [10,11,12,13,16]. To date, the economic and social impact of recurrent PSP, especially due to hospital readmission, is rarely reported.

In a previous study, Schramel FM et al. [17] analyzed the cost effectiveness of VATS versus conservative treatment for first time or recurrent spontaneous pneumothorax. They reported VATS to be cost effective and associated with less morbidity compared to conservative therapy. In another study, Torresini G et al. [18] reported a cost reduction due to VATS in patients with the first episode of spontaneous pneumothorax compared to CT treatment. Moreover, in a recent meta-analysis of Daemen JHT et al. [14], VATS was reported to be associated with significantly reduced ipsilateral recurrence rates and shorter length of hospitalization compared to CT treatment.

In our study, 48 patients were initially treated with CT. In this group, persistent air leak for more than 5 days was evident in 11 patients (22.9%), of whom 10 (20.8%) had to receive VATS during first hospital admission. The primary treatment cost in the CT group, including hospitalization and surgical material, was calculated at EUR 1.247 (Table 4). The total management cost of the 10 patients who underwent secondary VATS for prolonged air leak was calculated at EUR 13.530 (Table 5). Recurrent pneumothorax occurred in 25 of the CT patients, who were subsequently operated on using VATS. The management cost for these 25 patients was assessed at EUR 33,825, or EUR 1.353 per patient (Table 3). Overall, the total management cost of primary and recurrence treatment for the CT group was thus calculated to be EUR 93.675, with a cost per patient of EUR 1.952 (Table 6). The total management cost in this group increased to EUR 2.233 per patient after adding treatment costs for complications (prolonged air leak).

Likewise, 48 patients in our study cohort underwent primary VATS (VATS group) upon presentation at our hospital. One patient (2.1%) suffered a hemothorax and was reoperated by VATS. This complication rate is in line with previous reports [17,19]. The overall treatment cost of this complication as calculated at EUR 1.353 (Table 5). Interestingly, the primary treatment costs per patient, including hospitalization and surgical material in the VATS group, was higher (EUR 1.360) compared to the CT group (EUR 1.247). This difference was due to the higher cost of surgical materials for VATS (EUR 465 vs. EUR 77) (Table 4). In contrast with the CT group, significantly fewer patients (*n* = 4, 8.3%) suffered recurrence in the VATS group, which is similar to recently published data [10,14]. These patients subsequently underwent re-VATS. Due to the low rate of recurrence, overall cost for recurrence treatment was lower in the VATS group (EUR 5.412) than in the CT group (EUR 33.825). This disparity resulted in a considerably lower total management cost per patient in the VATS group (EUR 1.473) compared with the CT group (EUR 1. 952) (Table 6). Interestingly, this disparity increased considerably after addition of the treatment cost for complications (VATS: EUR 1.501 vs. CT: EUR 2.233; Table 6).

In this study, the mean LOS before and after treatment for recurrence was similar in both groups (Table 2 and Table 3). The main causes for the increased economic burden in the CT group were the high recurrence rate and the high rate of persistent air leaks after CT treatment, both requiring secondary VATS. This resulted in an overall 48.8% increase (EUR 732 per case) in total management and complication cost of the CT group versus the VATS group.

This study has limitations, primarily due to its retrospective nature and lack of randomization. Therefore, future prospective randomized controlled trials with a large number of patients are needed. Secondarily, the calculated costs are those specific to our clinic. These costs may vary (especially due to varying surgical material costs and the cost of a hospital stay) between hospitals in Germany and internationally.

Furthermore, there might be a selection bias due to the various indications for immediate surgery (in the VATS group, not all the patients underwent surgery for the same reason). Lastly, the treatment modality we used might not be the same in other countries, e.g., observation or needle aspiration instead of chest tube treatment, as described in previous studies. However, this study displays significant results concerning the socioeconomic impact of both treatment modalities in PSP patients. These results should not be considered to compare the effectiveness of VATS and CT treatment for a first episode of PSP, but can be used to compare their socioeconomic impact on patients and the healthcare system.

## 5. Conclusions

In conclusion, performing VATS upon first PSP seems to be associated with reduced socioeconomic burden, not only for the patients, but also for the healthcare system.

## Figures and Tables

**Table 1:** Demographics.

Variables	VATS (*n* = 48)	Chest Tube (*n* = 48)
Gender		
Male	39 (81.3)	40 (83.3)
Female	9 (18.7)	8 (16.7)
Age (mean)	24.8 (range 18–39)	26.2 (range 18–40)
BMI (kg/m^2^)	20.2	21.1
Height (cm)	180	180
Weight (kg)	65.5	70

Unless otherwise specified, all data are presented as the mean (range) and are based on the total patient cohort (*n* = 48 per group). BMI: body mass index; VATS: video-assisted thoracoscopic surgery.

**Table 2:** Postoperative parameters.

Variables	VATS (*n* = 48)	Chest Tube (*n* = 48)	*p*-Value
Duration of chest tube (days)	5	6	0.06
Mean LOS (days)	6	6	1.00
Prolonged air leak > 5 days (*n*)	6 (12.5%)	11 (22.9%)	0.181
Operation due to prolonged air leak *	0 (0%)	10 (20.8%)	0.001 *
Hemothorax (*n*)	1 (2.1%)	0 (0%)	0.315
Recurrence during follow-up (*n*) *	4 (8.3%)	25 (52.1%)	<0.001 *

Unless otherwise specified, all data are presented as mean and are based on the total patient cohort. * *p*-value < 0.05 displays statistical significance. LOS: length of hospital stay; VATS: video-assisted thoracoscopic surgery.

**Table 3:** Management costs of recurrence.

Variables	VATS (*n* = 4)	Chest Tube (*n* = 25)
Mean LOS at recurrence (days)	6	6
Duration of chest tube (days)	5	5
Cost of hospital day, as per DRG	EUR 148	EUR 148
Total LOS costs (pp/pg)	EUR 888/EUR 3.552	EUR 888/EUR 22.200
Surgical material cost (pp/pg)	EUR 465/EUR 1.860	EUR 465/EUR 11.625
Recurrence treatment costs (pg)	EUR 5.412	EUR 33.825
Recurrence treatment costs (pp)	EUR 1.353	EUR 1.353

VATS: video-assisted thoracoscopic surgery. All patients received VATS or re-VATS depending on their initial treatment. pp = per patient; pg = per group; LOS: length of hospital stay; DRG: diagnosis related group. The cost of surgical materials and cost of a hospital stay per day may differ across hospitals.

**Table 4:** Primary management costs.

Variables	VATS (*n* = 48)	Chest Tube (*n* = 48)
Mean LOS (days)	6	6
Cost of hospital day, as per DRG	EUR 148	EUR 148
Total LOS costs (pp/pg)	EUR 888/EUR 42.624	EUR 888/EUR 42.624
Surgical material cost (pp/pg)	EUR 465/EUR 22.320	EUR 77/EUR 3.696
Primary treatment costs (pp)	EUR 1.360	EUR 1.247
Primary treatment costs (pg)	EUR 65.288	EUR 59.850

pp = per patient; pg = per group; LOS: length of hospital stay; DRG: diagnosis-related group; VATS: video-assisted thoracoscopic surgery. The cost of surgical materials and the cost of a hospital stay per day may differ across hospitals.

**Table 5:** Complication management costs (secondary VATS).

Variables	VATS (Hemothorax, *n* = 1)	Chest Tube (Prolonged Air Leak, *n* = 10)
Mean LOS (days)	6	6
Cost of hospital day, as per DRG	EUR 148	EUR 148
Total LOS costs (pp/pg)	EUR 888/EUR 888	EUR 888/EUR 8880
Surgical material cost (pp/pg)	EUR 465/EUR 465	EUR 465/EUR 4650
Treatment costs (pp)	EUR 1.353	EUR 1.353
Treatment costs (pg)	EUR 1.353	EUR 13.530

VATS: video-assisted thoracoscopic surgery. All patients received VATS or re-VATS depending on their initial treatment. pp = per patient; pg = per group; LOS: length of hospital stay; DRG: diagnosis-related group. The cost of surgical materials and cost of a hospital stay per day may differ across hospitals.

**Table 6:** Total management costs.

Variables	VATS (*n* = 48)	Chest Tube (*n* = 48)
Primary treatment costs (pg)	EUR 65.288	EUR 59.850
Recurrence treatment costs (pg)	EUR 5.412	EUR 33.825
Total management costs (pg)	EUR 70.700	EUR 93.675
Total management costs (pp)	EUR 1.473	EUR 1.952
Total management and complication costs (pg)	EUR 72.053	EUR 107.205
Total management and complication costs (pp)	EUR 1.501	EUR 2.233

VATS: video-assisted thoracoscopic surgery; pp = per patient; pg = per group. The cost of surgical materials and cost of a hospital stay per day may differ across hospitals.

## Data Availability

The data presented are included in this study; additional data may be provided by the corresponding author on request.

## References

[1] Schnell J., Beer M., Eggeling S., Gesierich W., Gottlieb J., Herth F.J., Hofmann H.-S., Jany B., Kreuter M., Ley-Zaporozhan J. (2018). Management of Spontaneous Pneumothorax and Post-Interventional Pneumothorax: German S3 Guideline. Respiration.

[2] Sahn S.A., Heffner J.E. (2000). Spontaneous pneumothorax. N. Engl. J. Med..

[3] Noppen M. (2003). Management of primary spontaneous pneumothorax. Curr. Opin. Pulm. Med..

[4] MacDuff A., Arnold A., Harvey J. (2010). Management of spontaneous pneumothorax: British Thoracic Society pleural disease guideline 2010. Thorax.

[5] Baumann M.H., Strange C., Heffner J.E., Light R., Kirby T.J., Klein J., Luketich J.D., Panacek E.A., Sahn S.A. (2001). Management of spontaneous pneumothorax: An American College of Chest Physicians Delphi consensus statement. Chest.

[6] Tschopp J.-M., Bintcliffe O., Astoul P., Canalis E., Driesen P., Janssen J., Krasnik M., Maskell N., Van Schil P., Tonia T. (2015). ERS task force statement: Diagnosis and treatment of primary spontaneous pneumothorax. Eur. Respir. J..

[7] Noppen M., Alexander P., Driesen P., Slabbynck H., Verstraeten A. (2002). Manual aspiration versus chest tube drainage in first episodes of primary spontaneous pneumothorax: A multicenter, prospective, randomized pilot study. Am. J. Respir. Crit. Care Med..

[8] Ayed A.K., Chandrasekaran C., Sukumar M. (2006). Aspiration versus tube drainage in primary spontaneous pneumothorax: A randomised study. Eur. Respir. J..

[9] Olesen W.H., Lindahl-Jacobsen R., Katballe N., Sindby J.E., Titlestad I.L., Andersen P.E., Licht P.B. (2016). Recurrent Primary Spontaneous Pneumothorax is Common Following Chest Tube and Conservative Treatment. World J. Surg..

[10] Olesen W.H., Katballe N., Sindby J.E., Titlestad I.L., Andersen P.E., Lindahl-Jacobsen R., Licht P.B. (2018). Surgical treatment versus conventional chest tube drainage in primary spontaneous pneumothorax: A randomized controlled trial. Eur. J. Cardio-Thorac. Surg..

[11] Sawada S., Watanabe Y., Moriyama S. (2005). Video-assisted thoracoscopic surgery for primary spontaneous pneumothorax: Evaluation of indications and long-term outcome compared with conservative treatment and open thoracotomy. Chest.

[12] Herrmann D., Klapdor B., Ewig S., Hecker E. (2016). Initial management of primary spontaneous pneumothorax with video-assisted thoracoscopic surgery: A 10-year experience. Eur. J. Cardio-Thorac. Surg..

[13] Aljehani Y.M., Almajid F.M., Niaz R.C., Elghoneimy Y.F. (2018). Management of Primary Spontaneous Pneumothorax: A Single-center Experience. Saudi J. Med. Med. Sci..

[14] Daemen J.H.T., Lozekoot P.W.J., Maessen J.G., Gronenschild M.H.M., Bootsma G.P., Hulsewé K.W.E., Vissers Y.J.L., De Loos E.R. (2019). Chest tube drainage versus video-assisted thoracoscopic surgery for a first episode of primary spontaneous pneumothorax: A systematic review and meta-analysis. Eur. J. Cardio-Thorac. Surg..

[15] Kim H., Shin H.-J., Kim S.-W., Hong J.-M., Lee K.S., Lee S.-H. (2017). Psychological Problems of Pneumothorax According to Resilience, Stress, and Post-Traumatic Stress. Psychiatry Investig..

[16] Divisi D., Di Leonardo G., Crisci R. (2015). Video-assisted thoracic surgery versus pleural drainage in the management of the first episode of primary spontaneous pneumothorax. Am. J. Surg..

[17] Schramel F.M., Sutedja T.G., Braber J.C., van Mourik J.C., Postmus P.E. (1996). Cost-effectiveness of video-assisted thoracoscopic surgery versus conservative treatment for first time or recurrent spontaneous pneumothorax. Eur. Respir. J..

[18] Torresini G., Vaccarili M., Divisi D., Crisci R. (2001). Is video-assisted thoracic surgery justified at first spontaneous pneumothorax?. Eur. J. Cardio-Thorac. Surg..

[19] Crisci R., Coloni G.F. (1996). Video-assisted thoracoscopic surgery versus thoracotomy for recurrent spontaneous pneumothoraxA comparison of results and costs. Eur. J. Cardio-Thorac. Surg..

